# Genetic defects in human azoospermia

**DOI:** 10.1186/s12610-019-0086-6

**Published:** 2019-04-23

**Authors:** Farah Ghieh, Valérie Mitchell, Béatrice Mandon-Pepin, François Vialard

**Affiliations:** 1 EA7404-GIG, UFR des Sciences de la Santé Simone Veil, UVSQ, Montigny le Bretonneux, France; 2 CHU Lille, Reproductive Biology Institute-Spermiologie-CECOS, Jeanne de Flandre Hospital, Lille, France; 3 EA4308 “Gametogenesis and Gamete Quality”, University of Lille, Lille, France; 4 UMR BDR, INRA, ENVA, Université Paris Saclay, Jouy en Josas, France; 5 Genetics Division, CHI de Poissy St Germain en Laye, Poissy, France

**Keywords:** Azoospermia, Genetic defects, Chromosome, Mutations, Polymorphisms, Epigenetics, Azoospermie, Anomalies génétiques, Chromosome, Mutations, Polymorphismes, Épigénétique

## Abstract

As with many other diseases, genetic testing in human azoospermia was initially restricted to karyotype analyses (leading to diagnostic chromosome rearrangement tests for Klinefelter and other syndromes). With the advent of molecular biology in the 1980s, genetic screening was broadened to analyses of Y chromosome microdeletions and the gene coding for the cystic fibrosis transmembrane conductance regulator (*CFTR*). Decades later, the emergence of whole-genome techniques has led to the identification of other genetic defects associated with human azoospermia. Although *TEX11* and *ADGRG2* defects are frequently described in men with azoospermia, most of the causal gene defects found to date are private (i.e. identified in a small number of consanguineous families).

Here, we provide an up-to-date overview of all the types of genetic defects known to be linked to human azoospermia and try to give clinical practice guidelines according to azoospermia phenotype. Along with homozygous mutations, polymorphisms and epigenetic defects are also briefly discussed. However, as these variations predispose to azoospermia, a specific review will be needed to compile data on all the particular genetic variations reported in the literature.

## Résumé

Comme pour beaucoup de maladies humaines, les analyses génétiques en cas d’azoospermie étaient initialement limitées à la réalisation d’un caryotype, conduisant au diagnostic de réarrangements chromosomiques comme pour le syndrome de Klinefelter ou autres syndromes. L’avènement de la biologie moléculaire, dans les années 1980, a permis l’élargissement du dépistage génétique à la recherche des microdélétions du chromosome Y et aux anomalies du gène CFTR (cystic fibrosis transmembrane conductance regulator). Il a fallu attendre plusieurs décennies et l’apparition des techniques d’analyses du génome entier pour que soit réalisée l’identification d’autres anomalies génétiques associés à l’azoospermie humaine. Si les anomalies des gènes *TEX11* et *ADGRG2* sont fréquemment décrites dans la littérature pour les hommes présentant une azoospermie, la plupart des altérations génétiques découvertes à ce jour sont privées, identifiées dans un petit nombre de familles souvent consanguines.

L’objectif dans cette revue est de fournir un aperçu actualisé de toutes les anomalies génétiques décrites dans la littérature et associées à l’azoospermie humaine tout en essayant de fournir des guides de conduite diagnostique en fonction du phénotype de l’azoospermie. En plus des mutations homozygotes et délétères, les polymorphismes et les défauts épigénétiques sont également brièvement abordés. Néanmoins, comme ces variations ne sont que de potentiels facteurs de prédisposition à l’azoospermie, une étude spécifique sera nécessaire pour compiler l’ensemble des données de la littérature pour chaque variant génétique.

The World Health Organization (WHO) considers infertility (defined as the inability to conceive after 12 months of sexual intercourse without the use of contraceptives) to be a major health concern. Indeed, infertility affects more than 50 million couples worldwide. In about half of these couples, infertility is of male origin [1].

Semen analysis can often reveal congenital or acquired causes of male infertility. These include quantitative and/or qualitative abnormalities in spermatogenesis, which therefore affect the sperm count, sperm mobility and/or sperm morphology. Azoospermia (defined as the total absence of spermatozoa in the ejaculate in two successive semen examinations) accounts for around 10% of cases of male infertility, and affects about 1% of the men in the general population [2–4]. This condition can be classified as non-obstructive azoospermia (NOA, associated with spermatogenesis failure), and obstructive azoospermia (OA, characterized by an obstruction in the seminal tract and normal spermatogenesis). Whereas NOA accounts for 60% of azoospermic patients, OA accounts for around 40% [5, 6].

In almost all cases of azoospermia, the combination of sperm extraction with in vitro fertilization (IVF) and intra-cytoplasmic sperm injection (ICSI) gives these patients an opportunity to father children [7]. A variety of sperm extraction modalities and techniques have been developed, depending on the type of azoospermia. In general, sperm retrieval from the testis or epididymis should be prescribed for azoospermic patients [8]. In patients with OA, percutaneous epididymal aspiration, open fine-needle aspiration, or open surgical procedures (such as microsurgical epididymal sperm aspiration (MESA)) [9, 10] are often used for sperm retrieval. Sperm is successfully retrieved in more than 95% of cases.

However, the clinical management of NOA is more challenging; not all patients have sperm in their testes, and seminiferous tubules with complete spermatogenesis are intermixed with tubules without any germinal cells. In men with NOA, the sperm retrieval rate is around 40 to 50%. As is the case for OA, various sperm extraction techniques have been developed for men with NOA. According to the literature, microdissection testicular sperm extraction (microTESE) in several areas of the testis may be associated with higher sperm retrieval rates and lower postoperative complication rates [11–15].

Three histological phenotypes for NOA can be defined on the basis of the TESE (testicular sperm extraction) results: hypospermatogenesis, Sertoli-cell-only syndrome (SCOS), and maturation arrest (MA) [16, 17]. Thus, TESE also provides information on the infertility phenotype and guides the choice of treatments.

Maturation arrest is defined as incomplete spermatogenesis in which germ cells fail to mature. The condition is subcategorized into early MA, with the presence of spermatogonia or spermatocytes only (i.e. pre-meiotic or meiosis-arrested germ cells) and late MA, in which spermatids can be detected (i.e. post-meiotic arrest). In SCOS, germ cells are completely absent in all seminiferous tubules; only Sertoli cells and Leydig cells can be seen in the seminiferous tubules and the interstitial tissue, respectively [18, 19]. Lastly, hypospermatogenesis is characterized by the presence of all types of germ cell (from spermatogonia to spermatozoa), albeit in small numbers [20]. The degree of this histological phenotype can vary from mild to severe. Although a purely testicular histological phenotype can be found, the mixed pattern, is most frequent observed in azoospermic patients [16].

The many etiologies underlying azoospermia fall into pretesticular, testicular and post-testicular categories (see for review [21, 22]). Pretesticular (central) causes of azoospermia are endocrine abnormalities, and include hypogonadotropic hypogonadism, hyperprolactinemia, and androgen resistance. In contrast, testicular etiologies are characterized by disorders of spermatogenesis inside the testes, such as varicocele-induced testicular damage, undescended testes, testicular torsion, mumps orchitis, gonadotoxic effects of medications, genetic abnormalities, and idiopathic causes. Most cases of NOA have a pretesticular or testicular cause. Lastly, post-testicular etiologies (due to ejaculatory dysfunction or genital tract outflow obstruction) are the major contributors to OA [23, 24]. In the present review, we will not discuss pretesticular etiologies because they correspond to central nervous system defects and not to genital tract disease. Indeed, de novo or familial chromosomal or gene abnormalities constitute well-established genetic causes of azoospermia.

Genetic testing in human azoospermia was initially restricted to karyotype analyses [25–27]. With technical progress, genetic screening has been broadened to the analysis of the gene coding for cystic fibrosis transmembrane conductance regulator (*CFTR*) in patients with OA [28, 29] and Y chromosome microdeletions in patients with NOA [30–33]. Over the last 5 years, emergence of whole-genome techniques has led to the identification of many other supposedly causal genetic defects – raising the question of which genetic testing techniques should be used to evaluate human azoospermia. Here, we provide an up-to-date overview of all the types of genetic defects known to be linked to human azoospermia, including (i) chromosome abnormalities, (ii) causative gene mutations in OA, (iii) causative gene mutations in NOA, (iv) polymorphisms and (v) epigenetic alterations (Table 1). The last two types of defect are described in less detail.

**Table 1:** Genetic abnormalities observed in cases of obstructive or non-obstructive azoospermia

Genetic abnormality	Type of azoospermia	Sterility phenotype	Reference
Chromosome abnormalities			
Klinefelter syndrome	Non-obstructive azoospermia	Variable	[31]
47,XYY		Variable	[38, 39]
46,XX		SCOS	[46, 47]
Chromosome rearrangements		Variable	[21]
Y chromosome microdeletions			
AZFa	Non-obstructive azoospermia	SCOS	[59]
AZFb		Meiotic arrest	[59]
AZFc		Variable	[59]
Gene mutations			
*CFTR*	Obstructive azoospermia	CBAVD	[70, 73]
*ADGRG2*		CBAVD	[75]
*PANK2*		CBAVD	[87]
*SLC9A3*		CBAVD	[86]
*TEX11*	Non-obstructive azoospermia	Meiotic arrest	[90, 92]
*DMC1*		Meiotic arrest	[93]
*DNAH6*		Meiotic arrest	[94]
*MAGEB4*		SCOS	[97]
*MCM8*		Unknown	[99]
*MEIOB*		Meiotic arrest	[94]
*MEI1*		Meiotic arrest	[105]
*NPAS2*		Unknown	[108]
*PSMC3IP*		Unknown	[110]
*SPINK2*		Post-meiotic arrest	[111]
*STX2*		Meiotic arrest	[112]
*SYCE1*		Meiotic arrest	[114]
*TAF4B*		Unknown	[116]
*TDRD7*		Post-meiotic arrest	[119]
*TDRD9*		Meiotic arrest	[122]
*TEX14*		Meiotic arrest	[94]
*TEX15*		Meiotic arrest	[127]
*XRCC2*		Meiotic arrest	[132]
*ZMYND15*		Meiotic arrest	[116]

*CBAVD* congenital bilateral absence of the vas deferens, *SCOS* Sertoli-cell-only syndrome

## Chromosome abnormalities

### Klinefelter syndrome (KS)

This syndrome was the first chromosomal abnormality to be linked to male infertility. It was first described in 1942 [34], and is the most common genetic etiology of human male infertility. The syndrome is caused by a 47,XXY karyotype [35]. The prevalence of KS is close to 2 per 1000 male births [36, 37]. Eighty percent of cases of KS have a nonmosaic 47,XXY karyotype, whereas the remaining 20% variously show higher-grade chromosome aneuploidies, a 46,XY/47,XXY mosaic, or a structurally abnormal chromosome X [38]. Mosaic KS patients are usually less severely affected than nonmosaic patients are, and few cases of spontaneous paternity have been reported [39, 40]. This situation is not specific to humans; a XXY karyotype is always associated with infertility in various domestic animals (mice, cats, dogs, pigs, cows, horses, etc.) [41–43].

The presence of two X chromosomes in a male leads to impaired spermatogenesis and the failure of meiosis because gametogenesis is only possible for 46, XY cells - explaining the presence of gametes in mosaic patients (see for review [44]). Although very few functional tubules may be present in men, focal spermatogenesis enabled the recovery of spermatozoa (using TESE) in almost 50% of cases in a study of 1248 patients; however, none of the tested parameters (including age, testis volume, and levels of FSH, LH and testosterone (T)) had predictive value [45].

In KS, degeneration of the seminiferous tubules starts well before puberty [46] and progresses throughout infancy [47]. A dramatic increase in degeneration frequently occurs at puberty, and often leads to the complete hyalinization of the seminiferous tubules in adulthood [48]. It was initially recommended to cryopreserve testicular tissue as soon as possible in those cases. However, it is now generally acknowledged that TESE in young boys with KS is questionable; germ cells loss probably occurs very early [49], and so may explain the poor results seen for adolescent testicular tissue banking [50].

### 47,XYY syndrome

This syndrome was first described in 1961 [51], and is associated with a predisposition to infertility ranging from a normal sperm count to azoospermia [52, 53] . In fact, the supernumerary Y chromosome is probably lost in the early stages of spermatogenesis in the great majority of XYY males [54–56], thus enabling normal spermatogenesis. However, the supernumerary Y chromosome persists in some XYY males, which results in asynapsed sex chromosomes at the pachytene stage [57, 58]. In this situation, only a trivalent configuration could achieve meiosis [59].

### 46,XX males

In more than 80% of cases, a 46,X,der(X)t(X;Y)(p22.3;p11.2) karyotype results from an unbalanced de novo X-Y translocation and then the translocation of SRY (sex-determining region of Y chromosome) to the X chromosome. In the remaining 20% of cases, the genetic defect concerns the human sex determination pathway. 46, XX patients often exhibit SCOS [60, 61]. A defect in the SOX9 pathway is most frequently described, with duplication, triplication or balanced chromosomal translocation that overlaps with the so-called *RevSex* dosage sensitive critical region on chromosome 17q24 [62]. Other defects (like SOX3 duplication [63] and RSPO1 point mutation [64]) are rare but are frequently associated with a syndromic clinical presentation.

### Chromosome rearrangements

By comparing infertile men with newborn children, it was found that patients with impaired spermatogenesis have a greater number of chromosome abnormalities and/or rearrangements [65, 66]. Depending on the population studied, the proportion of affected individuals ranged from 2 to 20% [67–69], and the frequency of infertility increased with the severity of the impairment in spermatogenesis. Furthermore, it appears that gonosome abnormalities (aneuploidy or balanced translocation) most often result in azoospermia, whereas balanced abnormalities in autosomes tend to result in oligozoospermia.

Chromosome rearrangement appears to impact spermatogenesis through meiotic arrest. Several putative explanations for this association have been suggested. The first hypothesis is based on evidence of an interaction between the human quadrivalent chromosome (the association between the chromosomes involved in the translocation, at the pachytene stage), the acrocentric chromosomes, and the XY body - all of which are located near to the nucleolus [70–72]. This leads to an impairment in meiotic sex chromosome inactivation. The second hypothesis relates to the silencing of crucial genes in segments close to the chromosome breakpoints (due to the frequent non-pairing of these autosomal segments) and thus asynapsis. This hypothesis has been confirmed in studies of male mice [73] and boars [74] bearing a translocation.

### Y chromosome microdeletions

Frequent observations of Y chromosome rearrangements and large deletions in azoospermic males have suggested that a particular region is required for meiosis (e.g. 46,X,i(Y)(p11); 46,X,r(Y)). Experiments with specific probes have identified various interstitial deletions [75, 76], and have enabled the definition of three regions: AZFa, AZFb, and AZFc (azoospermia factor a, b and c) [77]. The prevalence ranges from 3 to 28%, depending on the type of impairment in spermatogenesis [78]. Although the AZFc phenotype is highly variable, full deletion of AZFa and AZFb always leads to azoospermia (SCOS, and pachytene MA, respectively) [79]. The complete deletion of AZFa and/or AZFb are currently the sole genetic abnormalities that contraindicate TESE.

Clinical practice: karyotyping and Y chromosome microdeletion screening are recommended by the latest international guidelines. This approach leads to a diagnosis in more than 15% of cases. Furthermore, a full AZFa and/or AZFb microdeletion diagnosis contraindicates a testicular biopsy.

## Causative gene mutations in OA

Some genetic diseases and abnormalities result in OA; they include cystic fibrosis, congenital bilateral absence of the vas deferens (CBAVD), congenital unilateral absence of the vas deferens, congenital bilateral epididymal obstruction and normal vasa, and Young syndrome. According to the literature, some gene mutations are associated with OA. We shall first describe *CFTR* mutations, and then mutations that have been described in the literature (starting with *ADGRG2* mutations).

### CFTR

This gene encodes a protein with an essential role in the sodium/chloride balance in cAMP-regulated epithelial secretions. Defects in the *CFTR* gene lead to the production of sweat with an abnormally high salt content and mucus secretions with an abnormally high viscosity. Complete loss of CFTR protein function leads to the autosomal recessive disease cystic fibrosis (CF) [80, 81]. The most common features of CF are respiratory symptoms, digestive problems, poor growth, short stature, and male sterility (due to CBAVD). The poor prognosis is due to bronchopulmonary involvement. To date, more than 2000 causal mutations are listed in public databases (https://www.re3data.org/repository/r3d100012093; [82]. These mutations are divided into different classes, depending on their effects on the protein and the disease mechanism [83–85]. Cystic fibrosis is the most common life-limiting genetic disorder in Caucasian populations. Several different explanations for the high frequency of heterozygotes in Caucasian populations have been suggested. Although greater fertility was initially hypothesized, it appears that heterozygosity for *CFTR* mutations confers greater resistance to typhoid fever [86], the effects of cholera toxin, and other diarrheal disorders [87]. Other hypotheses include (i) the development of cattle pastoralism, based on similarities in the distributions of lactase persistence and the most common CF mutation (Delta F508) [88], and (ii) possible respiratory advantages during the dusty climate of the last ice age [89].

Cystic fibrosis is caused by the presence of severe mutations (such as ∆F508, the most frequent *CFTR* mutation in Caucasian population) in both copies of the *CFTR* gene. This 3 bp deletion leads to the failure of CFTR protein to migrate to the plasma membrane [90]. Nevertheless, combinations of severe/mild mutations and mild/mild mutations lead to CFTR dysfunction that does not meet the diagnostic criteria for CF. These CFTR-related mutations are linked to a “minimal” phenotype that features CBAVD, chronic or recurrent acute pancreatitis, and disseminated bronchiectasis [91].

The incidence of CBAVD is as high as 6% in men with OA [92, 93]. The production of thick mucus in the genital tract associated with the CFTR mutations leads to vas deferens deterioration. Almost 80% of patients with CBAVD carry a *CFTR* mutation [94], and other etiologies might account for the phenotype in the remaining 20% of cases. Recently, a few genes have been linked to CBAVD as listed below.

Many studies have found a connection between *CFTR* mutations and impaired spermatogenesis [95]. A body of clinical evidence has highlighted an elevated mutation frequency and/or abnormally low expression of the *CFTR* gene in men with sperm abnormalities. The CFTR protein seems to be involved in spermatogenesis in rodent Sertoli cells and germ cells, and low CFTR protein expression has been observed in men with NOA [96]. Furthermore, CFTR has a critical role in sperm capacitation by directly or indirectly mediating HCO_3_^−^ entry, which is essential for this process [97].

### ADGRG2

**Fig. 1:**
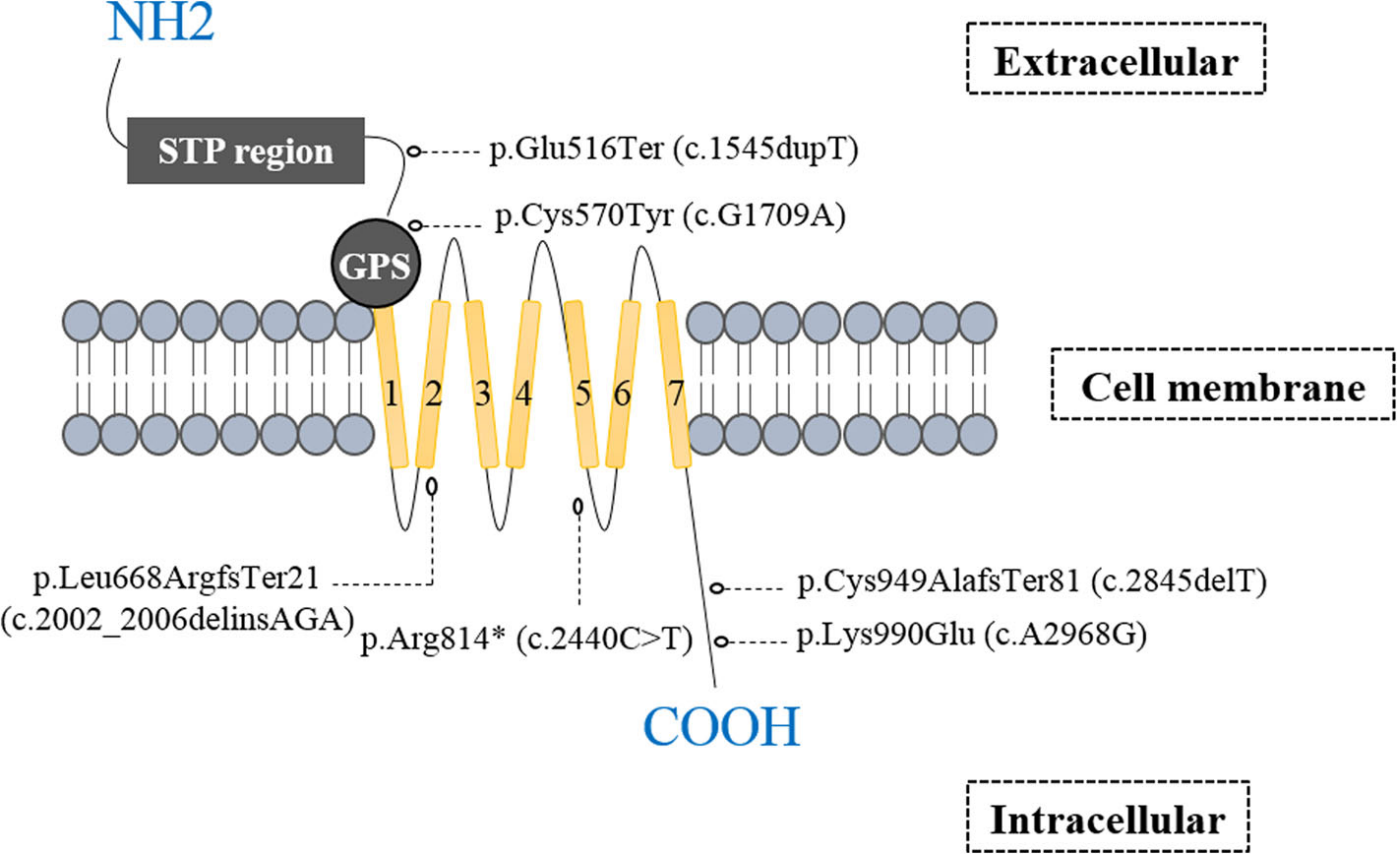
Schematic depiction of the structure of the ADGRG2 protein. Truncating mutations reported in OA patients are indicated. Yellow rectangles represent the seven transmembrane helices. ADGRG2 is also composed of a G-protein-coupled receptor (GPCR) autoproteolysis-inducing (GAIN) domain containing a cysteine-rich GPCR proteolysis site (GPS), and an extracellular STP region (in grey)

In patients lacking CFTR mutations, hemizygous protein-truncating mutations in the X-linked gene coding for adhesion G-protein-coupled receptor G2 (*ADGRG2*) were first described in a study of 26 azoospermic men [98], and then in a replication study of an unrelated population of 18 men [98, 99]. ADGRG2 (located in Xp22.3) is expressed in the efferent ducts and epididymis [100]. Moreover, ADGRG2 regulates fluid reabsorption in the efferent ducts through the ADGRG2-Gq/β-arrestin-1/CFTR signaling complex [101–103]. All patients with *ADGRG2* mutations (Fig. 1) displayed CBAVD only, and no other symptoms of CF (as with certain mild CFTR variants) - indicating a possible similar involvement of both genes in the development of CBAVD [98, 99] In a recent study, an additive nonsense *ADGRG2* mutation was described in two brothers with OA from a Pakistani family [104], confirming the involvement of *ADGRG2* mutations in OA.

### SLC9A3

The gene coding for solute carrier family 9 member A3 has also been described as pathogenic in patients with CBAVD. This protein is a Na+/H+ exchanger expressed on the apical membranes of cells in many structures (including the epididymis, vas deferens, and the non-ciliated cells of the efferent duct) [105, 106]. In the male reproductive tract, SLC9A3 is involved in fluid absorption and acidification [107]. It has been reported that loss of SLC9A3 decreases the expression of CFTR protein and causes OA in mice [108]. These findings suggest that SLC9A3 deletion has an impact in patients with CBAVD [109]. Further studies of the SLC9A3 gene’s involvement in CBAVD are required.

### PANK2

In a study of gene copy number variations in Asian patients with CBAVD [110], Lee et al. observed the homozygous loss of the *PANK2* gene encoding pantothenate kinase 2. This enzyme is the first in the co-enzyme A (CoA) biosynthetic pathway, and catalyzes the ATP (adenosine triphosphate)-dependent phosphorylation of pantothenate. Homozygous male mutants were infertile due to azoospermia [111] but also displayed retinal degeneration with progressive photoreceptor decline. The putative association between CBAVD and PANK2 has not been confirmed to date.

Clinical practice: given that almost 80% of patients with CBAVD carry a CFTR mutation [94], the latter gene should be fully sequenced. If a CFTR mutation is diagnosed, the patient’s spouse should also be tested (given the likelihood of CF in the offspring). If a CFTR mutation is not revealed by full sequencing, the patient could be screened for a possible defect in ADGRG2 – even though this diagnosis would not modify clinical practice.

## Causative gene mutations in NOA

The above-listed chromosome defects are observed in 15% of cases of azoospermia. Hence, one can reasonably hypothesize that most of the genetic causes of male infertility have yet to be characterized - probably because of the large number of genes involved [112]. Given that no more than 20% of men with NOA have chromosomal abnormalities, other spermatogenesis-related gene mutations are probably located elsewhere on the genome. To date, gene mutations have been discovered through studies of inbred families, which have confirmed the great genetic heterogeneity of this pathology. Furthermore, many azoospermic murine models have been described in the literature. A large number of possibly causal single-gene mutations have been reported for patients with the testicular phenotype of NOA (Table 1). Below, we briefly profile a number of candidate genes as a function of the testicular phenotype. We first describe mutations in *TEX11* (the gene most frequently cited in the literature) and then list other genes in alphabetical order.

### TEX11

**Fig. 2:**
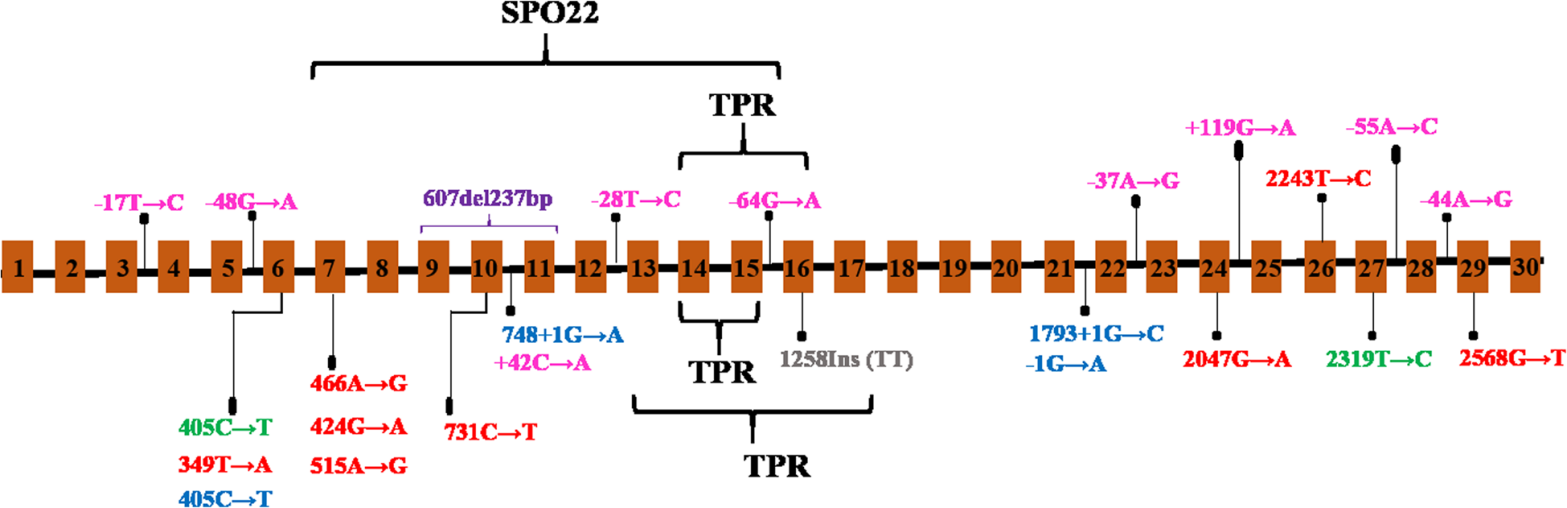
Schematic diagram of the location of TEX11 variants in isoform 2, as detected in patients with azoospermia. Brackets indicate the TEX11 protein’s interaction domains (the SPO22 sporulation domain and the TPR tetratricopeptide repeat-containing domain), according to the TEX11–203 transcript in the Ensembl database (https://www.ensembl.org/index.html). Orange boxes represent exons, and black lines represent introns. Missense mutations are shown in red, with splice site mutations in blue, silent mutations in green, frameshift mutations in grey, intronic mutations in pink, and deletions in purple

This gene (coding for testis expressed 11) on Xq13.1 appears to be the prime gene of interest in NOA. Initially, a 90 kb deletion (encompassing exons 9, 10, and 11) in one isoform of TEX11 was identified (using a chromosome micro-array) in two azoospermic patients with homogeneous or mixed meiotic arrest [113]. This deletion resulted in the loss of 79 amino acids from the TEX11 protein’s meiosis-specific sporulation (Spo22) domain. Additional *TEX11* mutations (missense and splice mutations) were found in 2.4% of the azoospermic patients. In line with the phenotype of male *Tex11*^−/−^ mice, a histological analysis evidenced meiotic arrest and low levels of TEX11 protein expression in patients bearing these mutations. The *TEX11* mutations reported to date (Fig. 2) are strongly associated with the occurrence of NOA due to testicular meiotic arrest [114]. In fact, TEX11 gene abnormalities are the sole defects recurrently described in the literature and in sporadic patients. The genes described below have been linked to azoospermia in consanguineous families.

### DMC1

DMC1 is essential for meiotic recombination in various organisms. Whole-exome sequencing of DNA (deoxyribonucleic acid) samples from two members of a consanguineous Chinese family (a man with NOA and a woman with premature ovarian insufficiency) enabled the identification of a homozygous missense mutation in the *DMC1* gene [115]. A detailed analysis evidenced MA at the zygotene stage in the seminiferous tubules of the patient with NOA.

### DNAH6

A rare, nonsynonymous mutation in the dynein axonemal heavy chain 6 (*DNAH6*) gene has been reported in azoospermic brothers from a consanguineous family [116]. DNAH6 protein is strongly expressed in testis, and *DNAH6* is important for meiosis [117] and ciliary beating. Mutations in *DNAH6* have also been linked to primary ciliary dyskinesia and sperm head anomaly [118], as well as to NOA.

### MAGEB4

The analysis of a consanguineous Turkish family led to the identification of a novel nonstop mutation in the X-linked gene *MAGEB4* (coding for melanoma antigen family B4) that segregated with an azoospermic and oligozoospermic phenotype [119]. In the testis, MAGEB4 is specifically expressed during germ cell differentiation [120].

### MCM8

[121] reported a homozygous mutation in the *MCM8* gene (coding for minichromosome maintenance complex component 8 and located on chromosome 20p12.3) in a consanguineous family in which a male with a 22q11.2 microdeletion presented azoospermia and a female had primary amenorrhea. Both individuals presented mild mental retardation. The complex formed by the MCM8 and MCM9 proteins has a key role in homologous-recombination (HR)-mediated DNA repair [122–124]. MCM8^−/−^ mice display infertility, a blockage in meiotic HR-mediated double-strand break (DSB) repair, and the absence of post-meiotic cells - confirming the importance of this gene in the meiotic stage of spermatogenesis [124].

### MEIOB

A homozygote non-synonymous mutation in the *MEIOB* gene has been identified in members of one family [116]. One of the brothers showed a meiotic arrest, as observed in *Meiob* knock-out mice [125, 126]. The mutation occurred in the MEIOB protein’s replication protein A1 DNA binding domain, and might have altered the meiotic recombination process. These studies highlight MEIOB’s role in meiosis (DSB repair and complete synapsis) and fertility in both humans and mice.

### MEI1

A homozygous missense mutation in the *MEI1* gene (coding for meiotic double-stranded break formation protein 1) has been described in two azoospermic brothers from a consanguineous family [127]. Meiotic arrest at the pachytene stage was confirmed in one brother. The mutation affecting the *MEI1* gene was found to co-segregate with the family’s NOA phenotype, and was heterozygous or absent in the other (fertile) family members. Meiotic double-stranded break formation protein 1 is overexpressed in testis, and is necessary for pairing of meiotic chromosomes. It may also be involved in the formation of meiotic DSBs in gonocytes. Mutant mice were infertile, due to meiotic arrest [128]. Consequently, defects in this gene are thought to disrupt the meiotic process. It has been reported that polymorphic alleles of the human *MEI1* are associated with human azoospermia caused by meiotic arrest [129].

### NPAS2

Using whole-exome sequencing, [130] identified a damaging non-synonymous mutation in *NPAS2* in three brothers with NOA from a consanguineous family. *NPAS2* (expressed in testis and cerebral cortex) encodes a member of the basic helix-loop-helix/PAS family of transcription factors, with functions in circadian rhythms and fertility.

### PSMC3IP

PSMC3 interacting protein has several functions, including the co-activation of ligand-dependent transcription mediated by nuclear hormone receptors, and the activation of DMC1 and RAD51 during meiotic recombination [131]. Recently, Al-Agha et al. identified a homozygous stop gain mutation in exon 6 of the *PSMC3IP* gene in an azoospermic man from a consanguineous family. This mutation was also present in his four sisters – all of whom suffered from primary ovarian insufficiency [132]. PSMC3IP is strongly expressed in testis of humans and mice. Null-mutant mice exhibit meiotic arrest at the spermatocyte I stage, and the failure of synaptonemal complex formation.

### SPINK2

SPINK2 is an acrosomal protein that targets acrosin in sperm and has an essential role in spermiogenesis. It is located in the acrosomal vesicle in round spermatids, and persists in mature spermatozoa. Researchers identified a homozygous splice mutation in the *SPINK2* gene in two brothers from a consanguineous family [133]. One of the two brothers had a low round spermatid count in a testicular biopsy. Studies of knock-out mice also confirmed the involvement of *SPINK2* in NOA, with spermiogenesis arrest at the round spermatid stage. This arrest was due to Golgi fragmentation and the failure of acrosome biogenesis in the absence of SPINK2 protein.

### STX2

Nakamura et al. identified a homozygous frameshift mutation in the syntaxin-2 (*STX2*) gene [134] in just one member of a population of 131 Japanese men with NOA. Histological analysis of the patient’s testis revealed MA and multinucleated spermatocytes. Furthermore, this gene is located within the 58.4 Mb genomic region with loss of heterozygosity, suggesting that the parents were consanguineous. In view of the phenotype seen in mice [135], it has been suggested that NOA may be caused by *STX2* mutations in a small proportion of patients.

### SYCE1

A pathogenic splice site mutation in the *SYCE1* gene (coding for synaptonemal complex central element 1) was identified in two azoospermic brothers with complete meiotic arrest from a consanguineous family [136]. This mutation disrupted the acceptor site of intron 3, and as a result, no SYCE1 protein could be detected in the patient’s seminiferous tubules. SYCE1 is one of the four components of the synaptonemal complex required for chromosome pairing. Its absence leads to the disruption of synapsis in mice [137].

### TAF4B

A homozygous mutation in the *TAF4B* gene (coding for TATA box-binding protein-associated factor 4B) resulted in NOA in two unrelated consanguineous families [138]. In the first family, the three affected brothers were homozygous for the same nonsense mutation in *TAF4B*; the resulting truncated protein lacked the histone fold domain (which is important for the DNA-binding activity of TAFs) and the TAF12 interaction domain). This gene is a transcriptional regulator enriched in human and mouse testis. However, *TAF4B* variants were not associated with NOA in a recent study of a Han population in north-east China [139]. Null mutant mice become infertile by the age of 3 months, with an absence of germ cells in the seminiferous tubules and an impairment in spermatogonial stem cell proliferation [140].

### TDRD7

A recent study of a consanguineous Chinese family reported two novel homozygous loss-of-function mutations in the *TDRD7* gene in individuals with congenital cataract and NOA [141]. One of the patients displayed a post-meiotic arrest in spermatogenesis, with the absence of mature spermatozoa in the seminiferous tubules. However, a *TDRD7* mutation is not a common cause for NOA because variants were not found in cohorts of patients with NOA alone or with congenital cataract alone. The researchers then confirmed the mutations’ impact in a mouse model, where the phenotype was similar to that seen in the two patients. *TDRD7* encodes a Tudor family protein required for the remodeling of dynamic ribonucleoprotein particles in chromatid bodies during spermatogenesis [142]. Furthermore, the encode protein repressed LINE1 retrotransposons in the male germline - highlighting its importance in spermatogenesis and male fertility.

### TDRD9

The Tudor-domain containing 9 protein (TDRD9) is a member of the DEAD-box helicase family. It represses transposable elements and prevents their mobility via the piwi-interacting RNA (piRNA) metabolic process [143]. A 4 bp deletion frameshift mutation in *TDRD9* has been identified in five infertile azoospermic men from a large consanguineous family; the mutation led to the loss of all the known functional domains [144]. *Tdrd9*^−/−^ male mice were sterile, with activation of retrotransposon line-1 and chromosomal synapsis failure [143].

### TEX14

*TEX14* is considered to be a novel causative gene for NOA because its expression is abnormally low in men with NOA [145]. TEX14 protein is exclusively expressed in testis, especially during meiosis [146]. *TEX14* has a major role in spermatogenesis, where it is thought to be required for the formation of intercellular bridges in germ cells during meiosis [147]. A recent study of two azoospermic brothers from a consanguineous family revealed a 10 bp frameshift deletion, which resulted in an early stop codon [116]. Azoospermia or infertility has also been observed in pigs [148] and mice with *Tex14* mutations [147].

### TEX15

In studies of two different families, mutations in the *TEX15* gene (required for meiotic recombination in spermatocytes) segregated with the NOA phenotype [149, 150]. In the first study, two brothers with NOA had a compound-heterozygote nonsense mutation. In the second, a homozygous nonsense mutation was identified in three Turkish brothers with azoospermia. Observations in a mouse model confirmed the patients’ infertility phenotype, since loss of the *Tex15* gene disrupted the DSB repair process and induced sterility (in males only) with meiotic arrest in the testis [151]. Two association studies of *TEX15* single-nucleotide polymorphisms (SNPs) gave contradictory results; a link to spermatogenetic failure was observed in one study [152] but not the other [153].

### XRCC2

Recently, Yang et al. identified a point mutation in the *XRCC2* gene (coding for X-ray repair cross-complementing protein 2 homolog, a RAD51 paralog) in two brothers with meiotic arrest and azoospermia from a consanguineous family [154]. The *XRCC2* gene’s product is involved in HR (homologous-recombination)-mediated DSB repair. Recreation of this mutation in mice using Crispr-Cas9 (clustered regularly interspaced short palindromic repeats associated proteins 9) technology also induced meiotic arrest and infertility, and thus confirmed its involvement in the patients’ phenotype. Another study identified a mutation in *XRCC2* that causes NOA and premature ovarian insufficiency [155]. One can therefore conclude that *XRCC2* is an essential for the progression of meiosis, and that a mutation in this gene could cause infertility in humans. Polymorphisms in XRCC2 homologs 1, 5, 6 and 7 have been linked to male infertility [156–158].

### ZMYND15

In three azoospermic brothers with MA at the spermatid stage, a homozygous mutation in the gene coding for ZMYND15 (zinc finger MYND-containing protein 15) led to amputation of the proline-rich domain (essential for cytoskeleton binding and signal transduction) [138]. ZMYND15 is involved in spermiogenesis and acts as a histone deacetylase-dependent transcriptional repressor. When ZMYND15 was inactivated, male mice displayed infertility and a low late spermatid count [159].

Clinical practice: with the exception of TEX11 defects (recurrent but rare in NOA), the other mutations seems to be private. So, whole-exome sequencing might be of diagnostic value, given that most gene defects are associated with meiotic arrest and thus rule out the retrieval of any spermatozoa. A number of points must to be considered: (i) the need for pedigree studies to identify consanguineous patients, (ii) the practical difficulty of analyzing genomic DNA samples, (iii) the time and cost of whole-exome sequencing, (iv) the absence of specific therapies, (v) the patient’s gratitude upon receipt of an etiologic diagnosis for his infertility. At present, whole-exome sequencing appears to have been restricted to clinical research. Hence, only TEX11 screening should be considered because defects are associated with meiotic arrest. However, the development of genetic analysis software and emergence of new genetic therapies (e.g. induced pluripotent stem cells [160]) might modify the diagnosis of NOA.

## Polymorphisms and related variations associated with azoospermia

Gene-targeted sequencing and candidate gene approaches have enabled the identification of a large number of SNPs and heterozygous mutations linked to azoospermia or which might predispose to impairments of spermatogenesis. Most of these studies were carried out on a small numbers of azoospermic patients and controls. We searched the PubMed database with the following keywords: (((((azoospermia[MeSH Major Topic]) or azoospermia[Title/Abstract]) AND (polymorphism[Title/Abstract] OR polymorphisms[Title/Abstract]))) NOT review[Publication Type]) NOT meta-analysis[Title]) AND English[Language], and then (((((azoospermia[MeSH Major Topic]) or azoospermia[Title/Abstract]) AND (mutation[Title/Abstract] OR mutation[Title/Abstract]))) NOT review[Publication Type]) NOT meta-analysis[Title]) AND English[Language]. The search yielded a list of more than 600 publications. After selecting only publications dealing with polymorphisms, SNP or heterozygote mutations, we found that 182 genes have been highlighted in azoospermic or oligo/azoospermic populations. The most frequently studied gene was *MTHFR*, in 19 different publications. Few genome-wide association studies have been performed in this field; a few loci have been identified but their association with male infertility has yet to be confirmed. We did not find any clear methodological proposals in the literature on how to use SNPs associated with spermatogenesis failure.

Clinical practice: screening polymorphism does not currently appear to be of great value because a diagnosis wouldn’t influence the patient’s treatment. Only MTHFR screening could be considered [161], despite the present lack of a randomized, placebo-controlled study.

## Epigenetic alterations in azoospermia

Along with genetic defects, epigenetic alterations (i.e. heritable alterations in gene function that do not affect the basic DNA sequence [162]) are now being increasing studied in the field of human infertility [163–166]. Epigenetics has an essential role during sperm production, sperm function, and fertilization. Sperm cells are epigenetically programmed through histone-protamine replacement, DNA methylation (> 80%), chromatin remodeling, genomic imprinting, and the involvement of small non-coding RNAs (piRNAs [167] and microRNAs (miRNA) [168, 169]). Hence, many studies have evidenced epigenetic changes in cases of azoospermia.

It was recently shown that mRNA and protein expression levels of the *KDM3A* gene (coding for lysine demethylase 3A) were abnormally low in testicular biopsies from patients with meiotic arrest at the round spermatid level or with SCOS, relative to samples from patients with OA [170]. Lysine demethylase 3A is a histone demethylase that is dynamically expressed in male germ cells. It regulates the expression of genes required for the packaging and condensation of sperm chromatin, such as *PRM1* and *TNP1* [166, 167, 171–173]. Furthermore, elevated histone H4 acetylation (essential for spermiogenesis) was observed in the nuclei of Sertoli cells in testicular biopsies from patients with SCOS, relative to controls [174]. Earlier, Sonnack et al. had observed low levels of H4 acetylation in the spermatids of patients with azoospermia; this contrasted with the hyperacetylation of this histone seen in spermatids from fertile patients [175].

In 2009, the methylation status of the promoter region of the *MTHFR* gene (coding for a regulatory enzyme involved in re-methylation reactions, DNA synthesis and the process of folate metabolism) was performed in patients with NOA and OA [176]. Relative to fertile controls, *MTHFR* was hypermethylated in DNA obtained from testicular biopsies (but not from peripheral blood) in men with NOA. It has been suggested that aberrant methylation of the *MTHFR* promoter reduces the expression and enzymatic activity of the encoded protein, leading to the development of azoospermia in these patients.

Genome-wide DNA methylation was subsequently assessed in testicular tissues from 94 azoospermic patients with OA or NOA and either positive or negative TESEs. The OA and NOA differed significantly with regard to the DNA methylation profile at over 9000 CpG sites. Accordingly, patients could be classified as having OA or NOA by considering the 212 CpG sites with the greatest methylation differences [177]. Fourteen of these 212 CpG sites were located in genes with a specific testicular function - suggesting the presence of epigenetic differences between types of azoospermia.

The association between DNA methylation and azoospermia has been extensively explored [178, 179]. For example, more than 30% of gene promoters differed in their DNA methylation status in men with NOA vs. fertile controls [180]. In particular, a hypermethylated *DDR1* gene (coding for discoidin domain receptor 1, a subfamily of receptor tyrosine kinases expressed in human postmeiotic germ cells) displayed an abnormal expression profile; it was overexpressed in 25% of the patients and underexpressed in 16%. The protein was not found in the testis of patients with SCOS.

Most recently, Li et al. have sought to identify methylation-regulated genes involved in NOA [181]. In a microarray analysis, a hypermethylated, down-regulated gene coding for zinc-finger CCHC-type containing 13 (ZCCHC13) was found to have low protein expression in NOA testis. The ZCCHC13 protein upregulates the AKT/MAPK/c-MYC signaling pathway. Hypermethylation of *ZCCHC13* might induce c-MYC lower expression and therefore act on cell differentiation and proliferation by altering the expression of c-MYC’s target genes.

Similarly, a study of the methylation status of the paternally imprinted *H19* gene and the maternally imprinted *MEST* gene in spermatogenic cells from azoospermic patients with either complete or incomplete MA revealed the presence of imprinting errors [182]. Low levels of H19 gene methylation were observed in primary spermatocytes and elongated spermatids, and MEST methylation errors were found in spermatocytes [182]. These results are in line with previous reports of gene imprinting errors in azoospermia [183].

These epigenetic alterations might be valuable biomarkers for male infertility in general and idiopathic azoospermia in particular. For example, it has been suggested that miRNAs (essential for spermatogenesis and possibly involved in the regulation of gene expression) are diagnosis biomarkers for azoospermia. Indeed, miRNA expression was altered in patients, relative to controls [179–182, 184–188]. A recent comparison of men with OA and men with NOA evidenced differences in miRNA expression in spermatogonia, spermatocytes and round spermatids, and thus suggested the presence of epigenetic dysregulation in NOA [189]. A comparison of subgroups of NOA patients with a positive vs. negative TESE gave similar results [190].

Clinical practice: in summary, it is clear that dynamic epigenetic processes are essential for normal spermatogenesis, and are being increasingly investigated in men with NOA. This research may open up perspectives for diagnosis and treatment.

## Conclusion

After the description of the Klinefelter syndrome karyotype (in 1959) and various chromosome rearrangements, it was several decades before the emergence of new genomic techniques initiated a new age for molecular studies of the etiology, mechanism, and diagnosis of azoospermia. Therapeutic approaches may even emerge in the near future. Genetic causes of azoospermia are not limited to gene alterations alone; epigenetic variations, SNPs and other polymorphisms have an impact on spermatogenesis.

Experiments in animal models will probably be needed to characterize all the pathways involved in spermatogenesis and (from a therapeutic perspective) circumvent defects in this process. New technologies (such as Crispr-Cas9) may make it possible to perform genome editing in animal models and thus confirm the causes of spermatogenesis failure.

Ideally, genetic studies of azoospermia should include a large number of patients with a defined phenotype, and a control group matched for ethnicity. Nevertheless, studies of consanguineous families may also generate new strategies that could be extended to all types of azoospermia.

Lastly, the following question arises; does it really make sense to restrict the genetic evaluation of azoospermia to karyotyping, *CFTR* testing and screening for chromosome Y microdeletions?

General guidelines:

Genetic screening in NOA: patients should be karyotyped and screened for Y chromosome microdeletions; these analyses lead to a diagnosis in more than 15% of cases, and contraindicate a testicular biopsy when a full AZFa and/or AZFb microdeletion is present. Depending on the geneticist’s experience, whole-exome sequencing could also be performed (together with a family segregation study). It should be borne in mind that guidelines on new gene defects are lacking, and that (with the exception of TEX11 defects) most gene defects are private.

Genetic screening in OA: with a view to avoiding CF in the offspring, patients with CBAVD should undergo whole gene sequencing. If mutations are detected, the patient’s spouse should also undergone this sequencing. Although screening might detect defects in ADGRG2, this observation would not change clinical practice.

## Abbreviations

ATP: Adenosine triphosphate
AZF: Azoospermia factor
CBAVD: Congenital bilateral absence of the vas deferens
CF: Cystic fibrosis
Crispr-Cas9: Clustered regularly interspaced short palindromic repeats associated proteins 9
DNA: Deoxyribonucleic acid
DSB: Double-strand break
HR: Homologous-recombination
ICSI: Intra-cytoplasmic sperm injection
IVF: In vitro fertilization
KS: Klinefelter syndrome
MA: Maturation arrest
MESA: Microsurgical epididymal sperm aspiration
micro-TESE: Microdissection testicular sperm extraction
miRNA: Micro RNA
NOA: Non-obstructive azoospermia
OA: Obstructive azoospermia
piRNA: Piwi-interacting RNA
SCOS: Sertoli-cell-only syndrome
SNP: Single-nucleotide polymorphism
TESE: Testicular sperm extraction
WHO: World Health Organization
